# Live-cell calcium imaging of adherent and non-adherent GL261 cells reveals phenotype-dependent differences in drug responses

**DOI:** 10.1186/s12885-017-3507-y

**Published:** 2017-08-02

**Authors:** Averey D. Strong, Richard L. Daniels

**Affiliations:** Department of Biology, The College of Idaho, Caldwell, ID 83605 USA

**Keywords:** Calcium imaging, Live cell imaging, Calcium Microfluorimetry, GL261, ATP, Capsaicin, Cell suspension, Neurosphere, Dissociated, Low melting point agarose

## Abstract

**Background:**

The tumor-derived GL261 cell line is used as a model for studying glioblastoma and other high-grade gliomas, and can be cultured adherently or as free-floating aggregates known as neurospheres. These different culture conditions give rise to distinct phenotypes, with increased tumorigenicity displayed by neurosphere-cultured cells. An important technique for understanding GL261 pathobiology is live cell fluorescent imaging of intracellular calcium. However, live cell imaging of GL261 neurospheres presents a technical challenge, as experimental manipulations where drugs are added to the extracellular media cause the cells to move during analysis. Here we present a method to immobilize GL261 neurospheres with low melting point agarose for calcium imaging using the fluorescent calcium sensor fura-2.

**Methods:**

GL261 cells were obtained from the NCI-Frederick Cancer Research Tumor Repository and cultured as adherent cells or induced to form neurospheres by placing freshly trypsinized cells into serum-free media containing fibroblast growth factor 2, epidermal growth factor, and B-27 supplement. Prior to experiments, adherent cells were loaded with fura-2 and cultured on 8-well chamber slides. Non-adherent neurospheres were first loaded with fura-2, placed in droplets onto an 8-well chamber slide, and finally covered with a thin layer of low melting point agarose to immobilize the cells. Ratiometric pseudocolored images were obtained during treatment with ATP, capsaicin, or vehicle control. Cells were marked as responsive if fluorescence levels increased more than 30% above baseline. Differences between treatment groups were tested using Student’s t-tests and one-way ANOVA.

**Results:**

We found that cellular responses to pharmacological treatments differ based on cellular phenotype. Adherent cells and neurospheres both responded to ATP with a rise in intracellular calcium. Notably, capsaicin treatment led to robust responses in GL261 neurospheres but not adherent cells.

**Conclusions:**

We demonstrate the use of low melting point agarose for immobilizing GL261 cells, a method that is broadly applicable to any cell type cultured in suspension, including acutely trypsinized cells and primary tumor cells. Our results indicate that it is important to consider GL261 phenotype (adherent or neurosphere) when interpreting data regarding physiological responses to experimental compounds.

**Electronic supplementary material:**

The online version of this article (doi:10.1186/s12885-017-3507-y) contains supplementary material, which is available to authorized users.

## Background

Glioblastoma multiforme (GBM) is the most common astrocyte-derived malignant brain tumor. Its prognosis is poor, with a median survival time of 15 months and a 10% survival rate 5 years post-diagnosis [1, 2]. Therefore, it is of fundamental public health interest to gain a better understanding of GBM in order to develop more effective treatments.

A number of primary tumor-derived cell lines serve as models for various aspects of glioma pathobiology [3, 4]. Among cell-based systems used to study high-grade gliomas such as GBM, the murine GL261 cell line displays important similarities to in vivo tumors. When implanted into syngeneic mice, GL261 cells often establish tumors that share many of the angiogenic and invasive properties characteristic of human GBM [2, 4–7]. Therefore the GL261 cell line has become a key model for investigating anti-tumor therapies and the underlying cellular mechanisms of tumorigenesis.

**Fig. 1:**
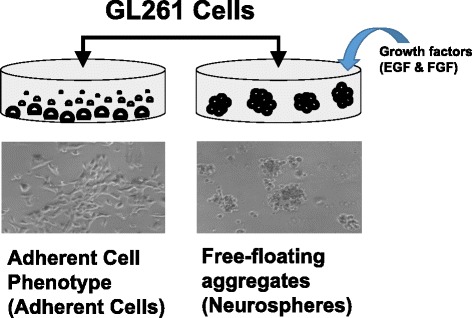
GL261 phenotype is dependent on culture conditions. GL261 cells grow adherently when cultured in media that contains serum. Cells cultured in serum-free media supplemented with EGF, FGF and B-27 grow as detached free-floating aggregates (neurospheres). When experimental manipulations involve acute drug treatments delivered to the media, live-cell fluorescent imaging of neurospheres presents a technical challenge as any treatment delivered to the culture medium causes cell movement

GL261 cells can be cultured in two different ways (Fig. 1). They can be grown as adherent cultures (GL261-AC) or, when cultured in the presence of growth factors, induced to differentiate and grow as free-floating aggregates called neurospheres (GL261-NS) [3, 8]. However, there are differences between the AC and NS phenotype, a finding consistent with primary cultures derived from human gliomas [9–11]. Mice implanted with GL261-NS cells survive on average 25 days, compared with 35 days for mice implanted with GL261-AC cells, and GL261-NS mouse tumors proliferate more rapidly in vivo than GL261-AC tumors. Additionally, real-time PCR and microarray analyses indicate that genes associated with processes such as neuronal differentiation, angiogenesis, and neurotransmitter transport are differentially expressed [9]. Taken together, these differences between GL261-AC and GL261-NS cells indicate the need for consideration of phenotype during pre-clinical testing of therapeutic compounds or other experimental manipulations.

Live-cell calcium imaging (calcium microfluorimetry) is a widely used method for monitoring acute responses to drug treatments and other experimental manipulations which elicit changes to intracellular signaling pathways that modulate cellular calcium [12–14]. This experimental paradigm has been applied to many cell types, including GL261 cells [15, 16]. However, for GL261 neurospheres (and other cell types that are cultured in suspension), live-cell imaging presents a technical challenge. When cells are not fixed to a substrate, any experimental manipulation (such as the introduction of a treatment solution into the media) causes the cells to move. This makes it difficult to compare pre- and post-treatment images and videos that permit analyses of calcium responses to external compounds in the GL261-NS phenotype.

A number of methods currently exist for immobilizing cells, tissue, and even whole organisms for live-cell fluorescent microscopy. These include embedding specimens in collagen, matrigel, methylcellulose, calcium alginate beads, low melting point agarose and other viscous substances [17–21]. Of these, low melting point agarose (LMPA) has a number of benefits as a laboratory reagent, including easy storage and relatively low cost (as compared with collagen and matrigel). Its use is well-documented for fluorescence imaging, and, in particular, for live-cell calcium imaging of whole organisms, tissue slices, and cells [22–29]. Furthermore, LMPA permits passive diffusion of solutes including glucose, lactate, and urea, at rates slower than water, but generally faster than collagen [30]. This permeability is important for understanding acute responses to membrane receptor agonists, chemotactic agents, and other compounds that bring about immediate changes to calcium-mediated cellular signaling events such as those detected by live-cell calcium imaging.

Here we present a method for immobilizing GL261 neurospheres in low melting point agarose (LMPA) for live-cell fluorescent imaging of calcium using the calcium sensor fura-2. Our method is compatible with experimental manipulations that rely on the diffusion of solutes, such as drugs infused into the extracellular solution. This technique extends previous methods by permitting microscopy-based analyses of individual GL261 neurosphere cells rather than aggregate cellular fluorescence data such as that obtained using fluorometry. Using this approach, we show that the GL261 adherent and neurosphere phenotypes are similar in their calcium responses to ATP, but not capsaicin (two agonists known to elicit intracellular calcium increases in GL261 cells) [15]. These data suggest that it is important to consider GL261 cell phenotype when interpreting data regarding cellular responses to experimental treatment compounds.

## Methods

### Cell culture

GL261 cells were obtained from the NCI-Frederick Cancer Research Tumor Repository (Frederick, MD). Adherent GL261 cells were cultured in DMEM (Corning) with 10% Fetal Bovine Serum (Atlanta Biologicals) and Penicillin/Streptomycin/Glutamine (Sigma-Aldrich). Cells were passaged by trypsinization and media was changed every 3–5 days. Neurosphere formation was induced by trypsinizing adherent GL261 cells and then culturing the cell suspension in the following media: serum-free DMEM with Penicillin/Streptomycin/Glutamine (Sigma-Aldrich), fibroblast growth factor (FGF-2, 20 ng/ml) (Peprotech), epidermal growth factor (EGF, 20 ng/ml) (Peprotech), and B27 (1:50) (Life Technologies). Differentiation was first noted 3–5 days after initial plating. Neurosphere culture media was replaced every 3–5 days by pelleting cells via centrifugation and re-suspending in new media. All cultures were maintained at 37.0 °C with 5.0% CO_2_ in 75 cm^2^ culture flasks (Nunc).

### Calcium microfluorimetry of adherent cells

Glass 8-well chamber slides (Thermoscientific Nunc Lab-Tek) were coated for 60–120 min with poly-L-lysine (Sigma-Aldrich). Adherent GL261 cells were trypsinized, plated in the 8-well chamber slides at 2 × 10^4^ cells/well, and allowed to incubate for 24 h before experiments. To prepare the cells for imaging, growth media was removed and the cells were washed twice with 200 μL of calcium imaging buffer (CIB; 130 mM NaCl, 3.6 mM KCl, 1.8 mM CaCl_2_, 1.0 mM MgCl_2_, 10 mM D-glucose, 10 mM HEPES, pH 7.4 adjusted with HCl and NaOH). After the final wash, 200 μL of 5 μM fura-2 (Invitrogen) in CIB was placed in each well. The cells were left to incubate for 30 min in the dark at room temperature. After incubation, wells were washed twice with 200 μL CIB and finally filled with 270 μL CIB. Test solutions were delivered by pipet while imaging: 30 μL of 2 mM ATP (Sigma; final concentration 200 μM), 30 μL of 1 mM capsaicin (Sigma; final concentration 100 μM), or 30 μL of the vehicle control (CIB) as treatments. Ratiometric pseudocolored images were captured with a Nikon Eclipse Ti-S epifluorescent inverted microscope equipped with a dual 340/380 nm excitation filter wheel (Sutter) and associated Nikon Digital Sight DS-U3 camera. Images and videos were analyzed using the Nikon Elements software package.

#### Calcium microfluorimetry of non-adherent cells (Neurospheres)

**Fig. 2:**
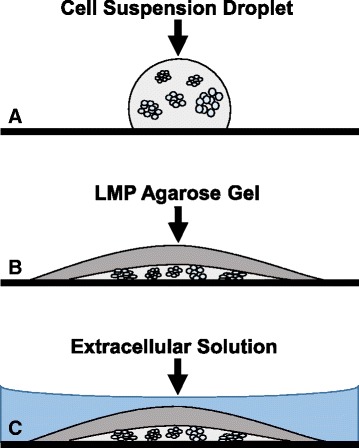
Immobilizing non-adherent GL261 cell neurospheres in low melting point agarose (LMPA) for live-cell fluorescent (calcium) imaging. **a** A 20 μL droplet containing suspended cells was applied to the bottom of the imaging 8-well chamber slide. **b** 20 μL of LMPA was placed on top of this droplet. LMPA is liquid at 37 °C (culture temperature of the cells) but solidifies upon cooling to 26–30 °C (room temperature at which imaging is performed). **c** Extracellular solution is placed on top of cells and LMPA. Test compounds can be delivered to the extracellular solution without disturbing cells, and solutes diffuse freely through the LMPA

For cells grown as neurospheres, the media containing the differentiated cells was placed directly into a 15 ml conical tube without need for trypsinization. The 15 ml conical tubes were centrifuged, supernatant was poured off, and the pellet of cells was re-suspended in 2 mL of CIB. This wash was repeated 2 times. After the final wash, cells were placed into 1 mL CIB containing 5 μM fura-2 for 30 min. During this time, a water bath was set to 40 °C and 15 ml of 0.5% low melting point agarose (LMPA, Bio-Rad #1620100) was made in a 50 mL beaker. The beaker was placed in the water bath and stirred intermittently during subsequent steps to limit solidification. An 8-well glass chamber slide (Thermoscientific Nunc Lab-Tek) was floated on top of the water in the water bath allowing it to equilibrate to the water temperature. After the 30 min fura-2 incubation, cells were centrifuged, and the pellet was re-suspended in 1 mL CIB. Centrifugation and re-suspension was repeated a total of 3 times. To fix the cells onto the 8-well chamber slide, 20 μL of cell suspension was first pipetted into each well in the floating 8-well chamber slide, followed by 20 μL of 0.5% LMPA to press and hold the cells against the bottom of the 8-well chamber slide (Fig. 2). The ends of pipet tips were sometimes cut off to allow for delivery of LMPA, which solidifies quickly upon cooling. The 8-well chamber slide was allowed to cool for 10 min at room temperature to ensure solidification of the agarose on top of the cells. CIB (270 μL) was added to each well carefully as to not disturb the cells and agarose layers. Test solutions and imaging were performed as described above.

### Analysis

In each image, 30 cells were randomly chosen prior to analysis and marked as regions of interest (ROIs) using Nikon Elements. Ratiometric imaging data were exported to a Microsoft Excel file and cells were marked as responsive if calcium rises resulted in a 340/380 ratio 1.3 times higher than the cells’ baseline reading. Each well was counted as an independent experiment. The percentage of responding cells out of the total was used for comparisons between treatment groups. The number of responding cells (rather than the response amplitude) were analyzed, as the average magnitude of the response depends greatly on the number of cells responding. Data were analyzed using Microsoft Excel 2013 and Sigma Plot 13. Differences between treatment groups were tested for significance with two-tailed Student’s t-tests or one-way analysis of variance (ANOVA).

## Results

### Adherent and neurosphere GL261 cells exhibit similar calcium responses to extracellular ATP

**Fig. 3:**
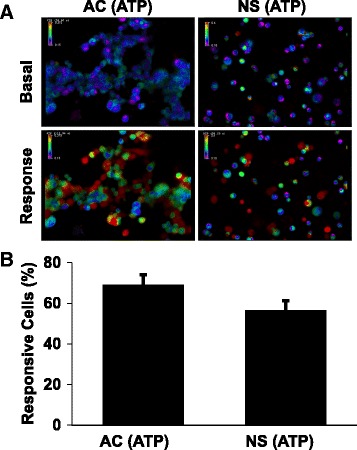
ATP evokes increases in intracellular calcium in both adherent and neurosphere GL261 cells. **a** Pseudocolored images represent intracellular calcium levels in adherent (AC) or neurosphere (NS) GL261 cells under basal conditions and after exposure to 200 μM ATP (200X magnification). Pseudocolor scale bars depict the fura-2 signal ratio, with warmer colors indicating increased intracellular calcium concentrations. **b** Mean percentage of AC (*n* = 11) or NS (*n* = 12) GL261 cells that respond to 200 μM ATP. Bars indicate standard error of the mean. No significant difference was detected between the two groups at a threshold of *p* < 0.05 (*p* = 0.09, Student’s t-test)

After establishing a method of using LMPA to immobilize non-adherent cells in our laboratory (see Methods and Fig. 2), we sought to test whether GL261 cells cultured under adherent (AC) or non-adherent (NS) conditions display differential calcium responses when treated with calcium channel agonists ATP and capsaicin (CAP). GL261 cells respond to extracellular ATP and capsaicin with an influx of calcium, and there is evidence that these responses are mediated by the ATP receptor P2X7 and the capsaicin receptor TRPV1, respectively [15, 31]. To test for a differential response, we used calcium microfluorimetry (live-cell calcium imaging) to observe intracellular calcium responses after 200 μM ATP or 100 μM CAP application. The concentration of ATP at the tumor border has been estimated to be in the hundreds of micromolar range in an animal model of ovarian cancer; thus 200 μM ATP approximates the physiological condition in some tumor microenvironments [32]. In addition, previous studies that have examined calcium entry and the cytotoxic effects of ATP on GL261 cells have used concentrations as high as 5 mM, finding ATP-induced calcium responses at 1 mM, but cytotoxic effects above this dose [15, 31]. The chosen ATP concentration therefore robustly induces a calcium response but is well below a cytotoxic dose. We found that both AC and NS cells display robust intracellular calcium rises in the presence of 200 μM ATP, as measured by a change in the fura-2 fluorescence signal ratio (Fig. 3; Additional file 1). No responses from AC or NS cells were observed when vehicle control (CIB) was added to the media (data not shown). We also found that the percentage of responding cells was similar between the adherent cells and neurospheres, as determined by a rise of 30% in the fura-2 fluorescence signal ratio. We found that in the adherent cells, 69.0% of all cells responded (*n* = 12 wells) and of the cells in neurospheres, 56.5% of all cells responded (*n* = 11 wells). No significant differences were observed when these treatment groups were compared with a two-tailed Student’s t-test (*p* = 0.09).

### Capsaicin responses are increased in neurospheres as compared with adherent GL261 cells

**Fig. 4:**
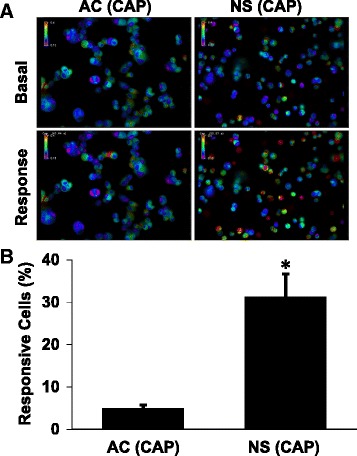
Capsaicin evokes a robust response in neurospheres but not adherent GL261 cells. **a** Pseudocolored images represent intracellular calcium levels in adherent (AC) or neurosphere (NS) GL261 cells under basal conditions and after exposure to 100 μM capsaicin (200X magnification). Pseudocolor scale bars depict the fura-2 signal ratio, with warmer colors indicating increased intracellular calcium concentrations. **b** Mean percentage of AC (*n* = 6) or NS (*n* = 9) GL261 cells that respond to 100 μM capsaicin. Bars indicate standard error of the mean. The difference between groups was significant (*) at a threshold of *p* < 0.05 (*p* = 0.0016, Student’s t-test)

After establishing that ATP responses were similar in adherent cells and free-floating cell aggregates (neurospheres), we tested whether different culture conditions affected capsaicin responses (Fig. 4; Additional file 1). We found that capsaicin (100 μM) elicited intracellular calcium rises in 31.3% of neurosphere cells (*n* = 9 wells), whereas only 5% of adherent cells displayed similar responses (*n* = 6 wells) as determined by a rise of 30% in the fura-2 fluorescence signal ratio. The difference between these treatment groups is significant, with a two-tailed Student’s t-test yielding a *p*-value of *p* = 0.0016.

### Acutely trypsinized cells display reduced calcium responses to ATP

Lastly, we tested whether acutely trypsinized adherent cells respond to ATP in a similar manner to cells that were cultured adherently. In some experimental protocols, such as isolation of particular cell types via flow cytometry and fluorescence activated cell sorting (FACS), it may be beneficial to perform live-cell calcium imaging on acutely trypsinized cells. However, researchers should note the effects of acute trypsinization on cellular calcium responses. We found that trypsinization reduces the number of cells responding to ATP to 30.8% of the total. When compared with the adherent cells and neurosphere treatment groups depicted in Fig. 3 with a one-way ANOVA, we found that a significant difference exists between the 3 treatment groups (*p* = 0.002). Post-hoc pair-wise comparisons (Holm-Sidak method) revealed that no significant differences were observed between the adherent and neurosphere cells (AC vs. NS, *p* = 0.104), but that the number of responding cells in the acutely trypsinized group was significantly decreased compared to each of the other two groups (AC vs. trypsinized cells, *p* = 0.001; NS vs. trypsinized cells, *p* = 0.024). This result indicates that calcium responses observed in adherent cells do not always directly correspond to calcium responses observed in acutely trypsinized cells.

Together, these results demonstrate a novel use of a LMPA-based approach for immobilizing GL261 cells, a method that can be applied to any cell type that is cultured in suspension or acutely trypsinized. Using this method, we find that calcium responses to ATP are similar between adherent cells and neurospheres, but that capsaicin responses differ based on phenotype.

## Discussion

Here we have described a method of immobilizing GL261 cells cultured as neurospheres in low melting point agarose. Using fluorescent imaging with the calcium sensor fura-2, we found that the phenotype of GL261 cells influences their physiological response to ATP and capsaicin. This work is consistent with previously reported effects of these compounds. Stock et al. reported that GL261-NS cells respond to capsaicin via the cation permeable channel TRPV1 with an increase in calcium based on fura-2 fluorescence in a fluorometer. This same report also indicated that ATP-induced calcium increases are observed in GL261-NS cells [15]. Another report indicates that trypsinized adherent cells exhibit acute calcium responses to ATP based on fluo-4 fluorescence in a fluorometer [16]. Tamajusuku et al. reported that adherent GL261 cells express P2X7, an ionotropic ATP receptor that presumably would lead to calcium influx upon ATP exposure [31]. Our work extends previous research by directly comparing calcium responses under the adherent and neurosphere culture conditions.

During the course of our experiments we made three observations regarding live cell calcium imaging of LMPA-embedded GL261-NS cells. First, we observed that there was variability in the timing of the cellular responses to ATP and capsaicin, instead of a nearly simultaneous response as is normally observed during imaging of adherent cells. Presumably this is because the diffusion of ATP and capsaicin varies by location throughout the agarose, and the drugs reach the cells at different times. Secondly, we observed that the thickness of the agarose has a direct correlation on the time it takes for diffusion to occur; that is, as the amount of LMPA used is increased, the cellular response to infusion of a compound is delayed, sometimes by up to 10 min. Specifically, we noted that after application of 20, 50, 100, and 150 μL of LMPA, that the time to drug response increased from 137.4 s to 157.1, 168.3, and 257.6 s, respectively (data not shown). Third, we found that acutely trypsinized adherent cells embedded in LMPA responded in lower numbers to ATP than adherent cells (Additional file 1). This could possibly be the result of disruption of extracellular proteins by trypsin. None of these observations presented an obstacle to imaging the cells; however a more rigorous quantification of these differences is needed to better understand cellular responses of GL261-NS cells.

## Conclusion

In summary, the present study adapts previously described methods for LMPA-based immobilization of cells to the GL261 model system. This method is broadly applicable to any cells cultured in suspension, or for primary cells from animal- or patient-derived tumors. Our results indicate that consideration of GL261 phenotype is warranted when interpreting data regarding calcium responses to experimental compounds. Improved live-cell imaging methods will be useful in further characterizing the pathobiology of glioblastoma and other high-grade glioma tumors.

## Additional file

Additional file 1: A table of the average number of responding cells for the reported treatments is supplied as supplementary data (Filename: Strong and Daniels – Data.xlsx). This file contains the observed average number of adherent and neurosphere cells responding to ATP (columns B and C), the average number of trypsinized adherent cells that responded to ATP (column D), and average number of adherent and neurosphere cells responding to ATP (columns E and F). (XLSX 11 kb)

## Abbreviations

AC: Adherent Cells
CAP: Capsaicin
GBM: Glioblastoma multiforme
LMPA: Low melting point agarose
NS: Neurospheres
TRPV1: Transient receptor potential vanilloid 1
